# Diabetic Ketoacidosis Updates: Titratable Insulin Infusions and Long-Acting Insulin Early

**DOI:** 10.1155/2021/1601553

**Published:** 2021-12-15

**Authors:** Justin Kinney, Oshin Baroi, Mania Gharibian

**Affiliations:** Department of Pharmacy Practice, Loma Linda University School of Pharmacy, 24745 Stewart St, Shryock Hall, Loma Linda 92350, CA, USA

## Abstract

**Background:**

To compare a titratable insulin infusion order set (vs. nontitratable) and early administration of long-acting insulin in adult patients with diabetic ketoacidosis (DKA).

**Methods:**

Single health system, retrospective study of adult patients admitted to the intensive care unit (ICU) for DKA. The primary outcomes were insulin infusion duration and ICU/hospital length of stays (LoS). Secondary outcomes included ICU/hospital survival, hypoglycemia, and hypokalemia.

**Results:**

151 patients were included in the titratable versus nontitratable insulin infusion comparison. Patients treated with the titratable insulin had shorter hospitalization (6.4 vs. 10.4 days, *p*=0.03) and reduced the number hypoglycemic events by over half (20.6% vs. 46.0%, *p* < 0.01). 110 patients were identified to compare overlapping a long-acting insulin for more than 4 h with the insulin infusion versus the standard 1-2 h overlap. Patients who received the insulin early spent over 18 h longer on the infusion (*p* < 0.01).

**Conclusions:**

A titratable insulin infusion added to the institutional DKA order set was associated with fewer days in the hospital and a significant reduction in hypoglycemic events. Furthermore, overlapping the long-acting insulin earlier with the insulin infusion early showed no benefit and could potentially be worse than the standard overlap.

## 1. Introduction

Diabetic ketoacidosis (DKA) is a life-threatening medical condition with acute metabolic complications generally requiring intensive care unit (ICU) admittance for higher level of care and correction of life-threatening acidosis. While it mainly occurs in patients with type I diabetes, it can develop in type II diabetics as well. Management of DKA focuses on the correction of metabolic acidosis, hyperglycemia, volume depletion, and electrolyte imbalances. This diabetic emergency is so common; many intuitions use standardized electronic order sets to streamline this process and improve patient care. The mainstay of therapy is a combination of regular IV insulin at a set rate of 0.1 units/kg/h (with ours without a bolus) and two-bag fluid system that is adjusted based on the patient's blood sugar, one fluid bag containing dextrose and one that does not [1, 2]. Insulin infusions are directly linked to patients staying in the ICU as a result of the hourly glucose checks, nursing staff demand, and high risk of adverse events. So, anything to decrease the necessary infusion time could result in shorter ICU and/or hospital length of stays yielding substantial cost savings. There is even exploration for mild cases of DKA to be managed with subcutaneous insulins alone outside of the ICU [3]. The order set at our health system changed from a nontitratable insulin infusion to titratable, adjusting the rate of insulin infusion based on the patient's blood sugars. Practitioners within the system were anecdotally reporting that the new titration led to prolonged ICU length of stay (LoS) and more time on the infusions as a result of the decreased insulin dose. Patients who appeared to having longer hospitalizations were those that the insulin was reduced to the lowest amount, 0.025 units/kg/h, overnight often resulting in a worsening acidosis by morning. This occurs because these patients require a minimum amount of insulin to be able to resolve the underlying ketoacidosis, despite normalized blood sugars, and such a small amount of insulin typically is not sufficient.

Hoping to decrease the duration of insulin infusions, a recent study in pediatrics showed potential benefit from the early administration of long-acting insulin during treatment the IV insulin infusion [4]. They found that the insulin combination aided in transitioning from IV insulin to subcutaneous as well as for discharge planning. This occurred when insulin glargine was overlapped for greater than 4 h with the infusion versus less than 2 h, standard practice being 1-2 hours. The therapy was well tolerated with similar incidences of hypoglycemia and only marginally higher rates of clinically insignificant hypokalemia. There is additional evidence supporting that early administration of long-acting insulin may lead to a quicker resolution of DKA, hospital duration, and help avoid hyperglycemic events after the drip discontinuation [4, 5]. The management of DKA has become fairly standardized in recent years, so we were hopeful that this novel intervention would yield similar benefits in the adult population, similar to what was seen in pediatrics.

The goals of this study were to evaluate the duration of insulin infusions and ICU/hospital length of stays in patients being treated for DKA as a result of two interventions: first, a titratable insulin infusion order set vs. nontitratable; and second, early administration of long-acting insulin while the insulin infusion is running vs. the standard 1-2 h overlap.

## 2. Methods

This was a single health system, retrospective analysis of adult patients diagnosed with DKA at Loma Linda University Health; the chart review period dated from 2012 to 2018.

The first intervention included patients who were over the age of 18 and admitted for DKA. The study arm included those who received the new titratable insulin infusion order set, released in October of 2016, while the control utilized the original nontitratable infusion. Patients who were pregnant were excluded. The new order set is built to be adjusted by nursing staff cutting the insulin infusion dose by 50% when blood sugars are between 200 and 250 mg/dl, followed by an additional 50% reduction when the sugars are less than 150 mg/dl; the two-bag system remained the same with titratable fluids. The insulin infusion initially is dosed at 0.1 units/kg/h leading to a dose of 0.05 units/kg/h and 0.025 units/kg/h after each of the titration. The two-bag fluid order set contains one fluid with dextrose and one without. The bag without dextrose runs alone at 250 mL/h, while the patient's sugars remain greater than 250 mg/dl, both bags run at 125 mL/h when the sugars range between 200 and 250 mg/dl; then, when they are less than 200 mg/dl, the bag with dextrose runs alone at 250 mL/h. Maintenance potassium is added to the two-bag system when it is less than 5.0 mmol/L.

The second intervention included patients over the age of 18 diagnosed with DKA and treated with long-acting insulin (insulin glargine) that overlapped with the regular insulin infusion for a minimum of 4 h, compared to the control arm who had their infusion overlap with long-acting insulin for less than 2 h, which is the standard. Patients were excluded if they were pregnant, receiving dialysis, or over the age of 80.

Loma Linda University Health granted IRB approval for this research, and data were extracted from patient's electronic medical record. Baseline characteristics reflect the first set of labs on admission. Hypoglycemic events were defined as low blood sugars requiring emergency dextrose administration, which occurs for a reading less than 70 mg/dl.

The primary outcomes were the duration of insulin infusion and LoS in the ICU/hospital. Secondary endpoints included ICU/hospital survival plus the incidence of hypoglycemia and hypokalemia. SPSS®, version 23, software was used for the statistical analyses. Descriptive statistics were compared using two-sided independent sample *t*-tests, while categorical variables were compared using two-sided chi-squared tests.

## 3. Results and Discussion

Approximately, 200 patients were screened for eligibility. For the titratable insulin infusion vs. nontitratable intervention, 64 patients were identified in the study arm and 87 in the control. Baseline characteristics are given in Table 1 with the nontitratable group being slightly more acidotic at baseline evident in their lactate, pH, anion gap, and bicarbonate. The duration of insulin infusion and length of stay in the ICU did not differ between the groups. Furthermore, there was no difference between ICU or hospital survival as well as incidence of hypokalemia. However, patients receiving the titratable insulin order set were hospitalized four days less on average relative to the nontitratable arm (6.4 days vs. 10.4, *p*=0.03) and had a 55% reduction in hypoglycemic events (20.6% vs. 46.0%, *p* < 0.01) (Table 2).

The second intervention exploring the combination of long-acting insulin early with the insulin infusion included 49 and 61 patients in the study and control groups, respectively. Baseline characteristics are given in Table 3 with the only difference being a higher percent of males in the control arm (26.5% vs. 55.7%, *p* < 0.01). The results showed no difference in ICU or hospital LoS or survival, plus similar safety profiles with comparable rates of hypoglycemia and hypokalemia. Unexpectedly, patients who received the long-acting insulin early experienced longer insulin infusion durations by nearly 18 hours (44.5 h vs. 27 h, *p* < 0.01) (Table 4).

Both interventions demonstrated results that were unanticipated, but of clinical significance when managing DKA patients. Practitioners regularly felt that the insulin drips were running longer because of the dose being titrated down overnight producing more acidotic patients in the morning. Time and energy are then wasted increasing the insulin dose and rebalancing the dextrose solution to begin correcting the acidosis again. But, the results of the study revealed the opposite was taking place in addition to a dramatic improvement in the need for emergency dextrose administration. The nontitratable arm was slightly more acidotic at baseline, but not by a clinically significant amount. Early administration of long acting insulin also exposed surprising results as those who received it were on the infusion substantially longer. This did not lead to longer length of stays, but did not demonstrate any benefit either and only adds potential risks from combining insulin therapies for extended amounts of time. The supporting literature available that spurred this research is small and focuses mainly on pediatrics, so at this time, it does not seem prudent to deviate from the standard 1-2 h overlap in adults.

## 4. Conclusions

The newest DKA order set at Loma Linda University is built in a titratable insulin infusion that allows nursing to adjust the dose based on the patient's blood sugar. In contrast to anecdotal reports from providers, this correlated with a shorter hospitalization as well as improved safety outcomes cutting the number of hypoglycemic events in half. Insulin infusions are high-risk orders, and the 50% reductions in its dose helped patient's maintain safe blood sugars while on it. The early addition of long-acting insulin to patients on an insulin infusion did not correlate with a decrease in its duration or improved LoS as previously cited and hypothesized. Therefore, we continue to support the standard practice of overlapping the therapies for 1-2 hours prior to the end of infusion. These results add to our advancing knowledge managing adult patients with DKA and expand beyond the standardized order sets in place at most institutions.

## Figures and Tables

**Table 1:** Titratable vs. nontitratable insulin infusion patients.

Baseline characteristics			
	Nontitratable (*n* = 87)	Titratable (*n* = 64)	*P* value
Age (years) mean (SD)	40.6 ± 19.9	43.6 ± 21.4	0.39
Sex (% male)	47.1	40.6	0.43
Weight (kg), mean (SD)	76.8 ± 22.0	76.6 ± 21.8	0.95
Lactate (mmol/L), mean (SD)	2.8 ± 2.4	2.2 ± 1.2	0.04
Creatinine (mg/L), mean (SD)	1.7 ± 1.7	1.3 ± 1.2	0.08
Dialysis (%)	9.2	4.7	0.30
Ventilated (%)	9.2	3.1	0.14
pH mean (SD)	7.25 ± 0.15	7.31 ± 0.10	0.01
A1C mean (SD)	11.6 ± 2.5	11.6 ± 2.8	0.98
Bicarbonate (mmol/L), mean (SD)	13.6 ± 7.4	16.3 ± 5.8	0.01
Serum ketones (mmol/L), mean (SD)	5.3 ± 3.7	4.4 ± 3.2	0.12
Anion gap, mean (SD)	27.1 ± 8.3	24.6 ± 5.9	0.03
WBC (bil/L), mean (SD)	14.1 ± 7.4	12.2 ± 5.3	0.06

SD, standard deviation; *n*, number.

**Table 2:** Titratable vs. nontitratable insulin infusion patients.

Results			
	Nontitratable (*n* = 87)	Titratable (*n* = 64)	*P* value
Primary endpoints			
Duration of insulin infusion (hours), mean	34.3	29.1	0.16
ICU LoS (days), mean	3.9	3.1	0.20
Hospital LoS (days), mean	10.4	6.4	0.03

Secondary endpoints			
ICU survival (%)	95.4	98.4	0.30
Hospital survival (%)	92.0	98.4	0.08

Safety endpoints			
Hypoglycemia (%)	46.0	20.6	<0.01
Hypokalemia (%)	70.1	62.5	0.14

*n* = number.

**Table 3:** Standard overlap vs. early addition of long-acting insulin to infusion.

Baseline characteristics			
	Standard (*n* = 61)	Early insulin overlap (*n* = 49)	*P* value
Age (years), mean (SD)	42.2 ± 21.2	35.4 ± 16.3	0.07
Sex (% male)	55.7	26.5	<0.01
Weight (kg), mean (SD)	75.6 ± 18.7	79.2 ± 25.6	0.40
Lactate (mmol/L), mean (SD)	3.0 ± 3.1	2.7 ± 1.7	0.58
Creatinine (mg/L), mean (SD)	1.3 ± 0.6	1.3 ± 0.9	0.74
Type 1 DM (%)	52.5	52.1	0.96
Ventilated (%)	11.5	6.1	0.33
pH mean (SD)	7.27 ± 0.1	7.27 ± 0.1	0.79
A1C mean (SD)	11.2 ± 2.4	12.1 ± 2.4	0.08
Bicarbonate (mmol/L), mean (SD)	13.1 ± 6.0	15.5 ± 7.8	0.07
Serum ketones (mmol/L), mean (SD)	5.2 ± 3.5	4.4 ± 3.4	0.25
Anion gap, mean (SD)	27.2 ± 8.2	25.1 ± 6.8	0.15
WBC (bil/L). mean (SD)	13.5 ± 7.2	13.9 ± 7.6	0.78

SD. standard deviation; *n*, number.

**Table 4:** Standard overlap vs. early addition of long-acting insulin to infusion.

Results			
	Standard (*n* = 61)	Early insulin overlap (*n* = 49)	*P* value
Primary endpoints			
Duration of insulin infusion (hours), mean	27	44.5	<0.01
ICU LoS (days), mean	3.2	4.1	0.27
Hospital LoS (days), mean	10.2	9.3	0.75

Secondary endpoints			
ICU survival (%)	95.1	93.9	0.78
Hospital survival (%)	90.2	93.9	0.48

Safety endpoints			
Hypoglycemia (%)	37.7	44.9	0.45
Hypokalemia (%)	68.9	75.5	0.44

*n* = number.

## Data Availability

The deidentified patient data are included within this article. Loma Linda University restricts sharing further patient specific information to protect patient privacy, and please contact the corresponding author with any additional questions.
